# Resistance training as a modulator of inflammation and cognition

**DOI:** 10.1080/15502783.2025.2550143

**Published:** 2025-08-22

**Authors:** Jacqueline Goodrich, Jamie L. Tartar, Jonathan Banks, Francesca Leon, Jose Antonio

**Affiliations:** a Nova Southeastern University, Department of Psychology and Neuroscience, Fort Lauderdale, FL, USA; b Nova Southeastern University, Exercise and Sport Science, Davie, FL, USA

**Keywords:** Hypertrophy, cognition, body composition, chronic inflammation

## Abstract

**Background:**

Inflammation is a natural immune response; however, prolonged or chronic inflammation has been implicated as a key contributor to chronic disease and cognitive decline. Research has shown that elevated levels of inflammatory biomarkers such as interleukin-6, C-reactive protein, and cortisol can impair cognitive function, highlighting the need for preventive measures. This study examines the effects of an 8-week resistance training program on peripheral markers of inflammation and cognitive function.

**Methods:**

This two-timepoint, within-subjects study will investigate the impact of an 8-week resistance training protocol on inflammation and cognitive performance. Participants *n* = 15 female (18.2 ± .36) untrained undergraduate students completed a baseline assessment, engaged in twice-weekly full-body resistance training, and underwent a post-training assessment. Baseline measures included cognitive performance tasks (Flanker Squared, Simon Squared, and Stroop Squared), saliva collection for IL-6, CRP, and cortisol analysis, body composition testing via InBody, a sleep quality index, and lifestyle assessment. The training protocol followed a progressive overload model to promote muscle hypertrophy, while emphasizing machine-based exercises under the researcher’s supervision to minimize injury risk due to the participants’ untrained status. After the 8-week resistance training program, the same assessments were repeated to identify changes. Statistical analysis included paired t-tests to examine the effects of resistance training on IL-6, CRP, and cortisol. Cognitive performance was analyzed via repeated measures t-tests for performance on the Neurotrax Cognitive Battery. Pearson correlations were also conducted to explore the relationship between biomarkers (CRP, IL-6, cortisol) and cognitive outcomes, with secondary analysis examining the impact of PSQI and lifestyle variables on the study’s results through within-subjects Pearson correlations.

**Results:**

Paired t-tests revealed significant improvements in cognitive performance: Flanker (*t*(14) = 5.27, *p* < .001), Stroop (*t*(14) = -4.61, *p* < .001), and composite cognition (*t*(14) = -5.11, *p* < .001), along with increased muscle mass (*t*(14) = -4.21, *p* < .001). Percent body fat was also found to be negatively correlated with changes in cognitive performance in composite cognitive performance (*r*(11) = -.63, *p* = .022) and Simon squared task scores (*r*(11) = -.62, *p* = .025). These findings suggest that resistance training may improve cognitive function and body composition, despite no measurable reductions the inflammatory biomarkers IL-6 and CRP.

**Conclusions:**

The results of this study support the beneficial effects of resistance training on cognition. More specifically, the 8-week resistance training program led to significant improvements in cognitive control, as demonstrated by increased scores on the Flanker & Stroop Squared tasks. These findings align with previous research indicating that physical exercise can be beneficial for cognitive processes. Furthermore, the observed negative correlation between body fat percentage and cognitive performance highlights the potential role and impact of body composition on cognitive health. These results indicate that resistance training could be an effective tool not only for improving physical health but also for supporting brain health, particularly in combating chronic inflammation.

